# Low-Dose Lactulose as a Prebiotic for Improved Gut Health and Enhanced Mineral Absorption

**DOI:** 10.3389/fnut.2021.672925

**Published:** 2021-07-27

**Authors:** Tarkan Karakan, Kieran Michael Tuohy, Gwendolyn Janssen-van Solingen

**Affiliations:** ^1^Department of Gastroenterology, Gazi University School of Medicine, Ankara, Turkey; ^2^Department of Food Quality and Nutrition, Research and Innovation Center, Fondazione Edmund Mach, San Michele all'Adige, Italy; ^3^Abbott Product Operations AG, Established Pharmaceuticals Division Headquarters, Allschwil, Switzerland

**Keywords:** lactulose, prebiotic, mineral absorption, short-chain fatty acid, SCFA, bifidobacteria, gut microbiota, gut health

## Abstract

Although medium and high doses of lactulose are used routinely for the treatment of constipation and hepatic encephalopathy, respectively, a wealth of evidence demonstrates that, at low doses, lactulose can also be used as a prebiotic to stimulate the growth of health-promoting bacteria in the gastrointestinal tract. Indeed, multiple preclinical and clinical studies have shown that low doses of lactulose enhance the proliferation of health-promoting gut bacteria (e.g., *Bifidobacterium* and *Lactobacillus* spp.) and increase the production of beneficial metabolites [e.g., short-chain fatty acids (SCFAs)], while inhibiting the growth of potentially pathogenic bacteria (e.g., certain clostridia). SCFAs produced upon microbial fermentation of lactulose, the most abundant of which is acetate, are likely to contribute to immune regulation, which is important not only within the gut itself, but also systemically and for bone health. Low-dose lactulose has also been shown to enhance the absorption of minerals such as calcium and magnesium from the gut, an effect which may have important implications for bone health. This review provides an overview of the preclinical and clinical evidence published to date showing that low-dose lactulose stimulates the growth of health-promoting gut bacteria, inhibits the growth of pathogenic bacteria, increases the production of beneficial metabolites, improves mineral absorption, and has good overall tolerability. Implications of these data for the use of lactulose as a prebiotic are also discussed.

## Introduction to Lactulose

### History and Clinical Use of Lactulose

**Figure 1:**
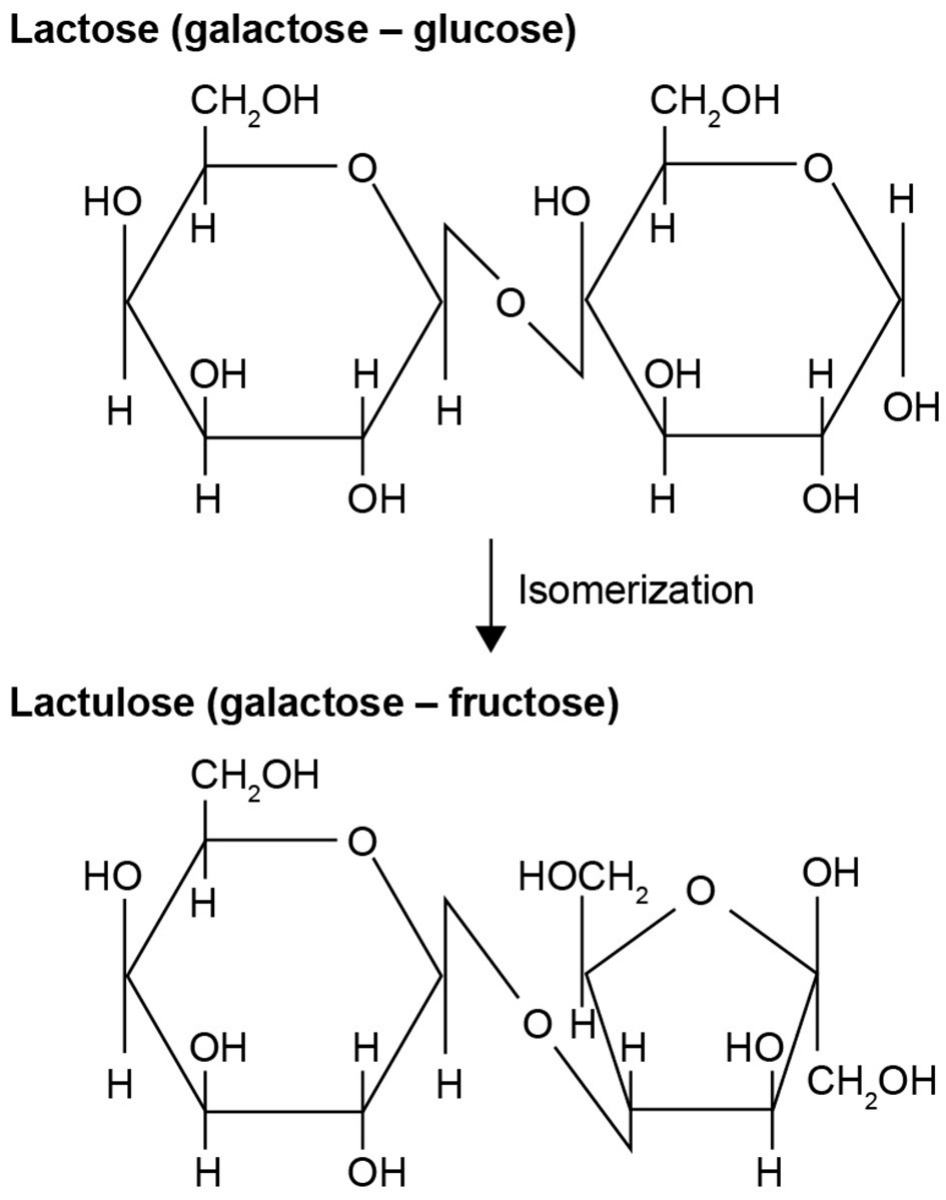
Chemical structure and formation of lactulose (1).

**Figure 2:**
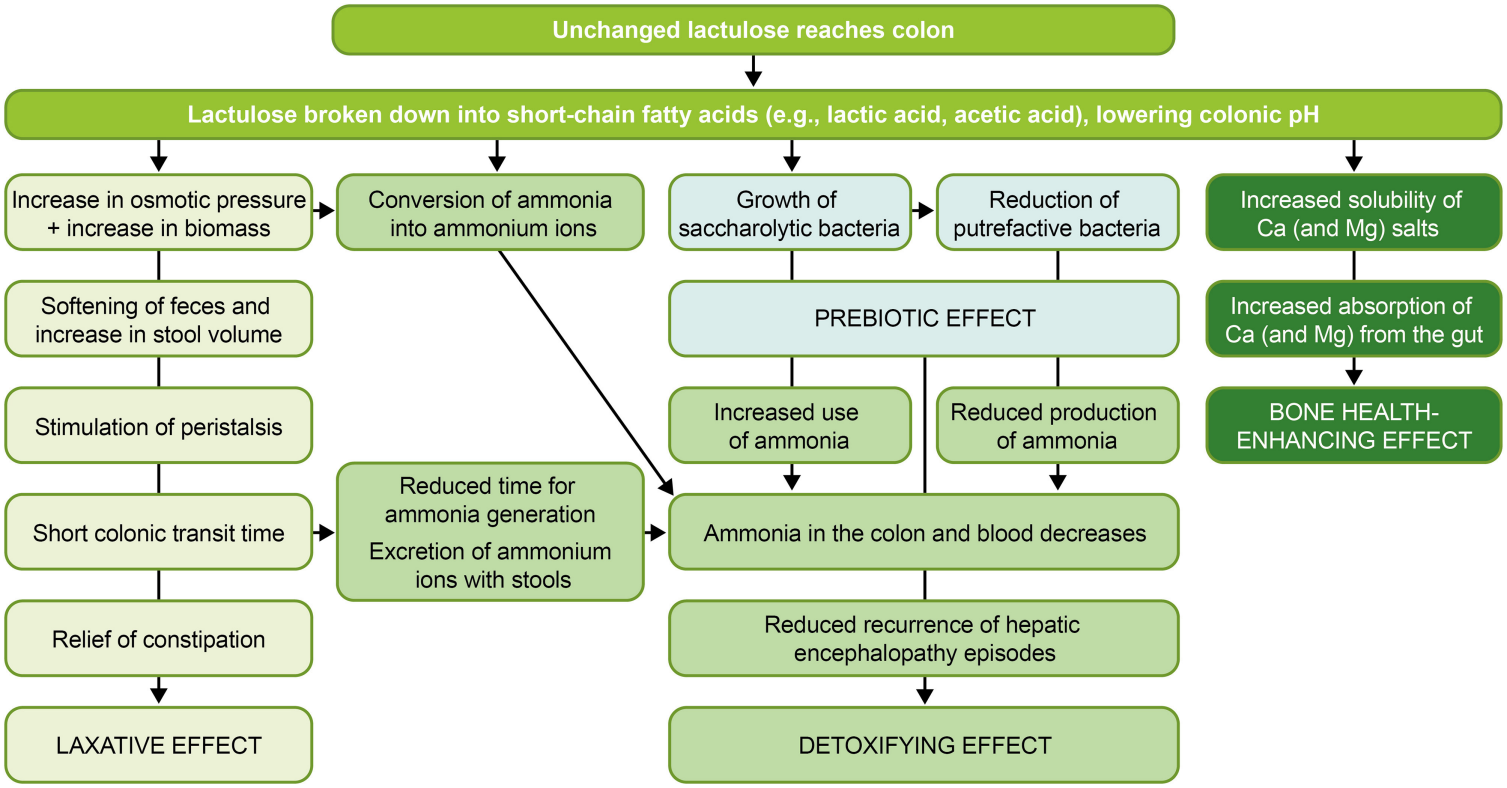
Mechanism of action of lactulose (1, 8–10). Ca, calcium; Mg, magnesium.

Lactulose is an artificial disaccharide composed of galactose and fructose, and is produced via isomerization of lactose (Figure 1) (1). Although first described by Montgomery and Hudson in 1929 (2), lactulose gained clinical interest only in 1957, when Petuely discovered that growth of fecal bacteria from the genus *Bifidobacterium* increased following administration of lactulose to infants (3, 4). Because of this activity (i.e., enhancement of bifidobacterial growth), Petuely referred to lactulose as “Der Bifidusfaktor” (“the bifidogenic factor”), a term still in use today (3). Based on the prebiotic and osmotic laxative properties of lactulose, Mayerhofer and Petuely proposed its use to treat constipation in 1959 (5), and lactulose has been used as a laxative for more than 50 years (6). In current clinical practice, lactulose is indicated as a laxative for the symptomatic treatment of constipation in children and adults and as a detoxifying agent for the treatment of hepatic encephalopathy (HE) in adults (Table 1 and Figure 2) (7). Although chiefly used for medicinal purposes at medium and high doses for the treatment of constipation and HE, respectively, low-dose lactulose can also be used as a prebiotic to stimulate the growth of health-promoting bacteria in the gastrointestinal (GI) tract, or gut (1, 11). Prebiotics such as lactulose are substrates that are selectively utilized by host microorganisms and that confer a health benefit (12). These can be non-digestible, short-chain carbohydrates that beneficially affect the host by selectively stimulating the growth and/or activity of one or a limited number of colonic bacterial species (13, 14). The numerous beneficial effects of prebiotics are summarized in Table 2.

**Table 1:** Lactulose clinical indications (7).

**Indication**	**Recommended dosing**
Constipation in children and adults	•Starting dose of 15–45 mL/day (10–30 g/day) and maintenance dose of 15–30 mL/day (10–20 g/day) in adults and adolescents. •Lower starting and maintenance doses are recommended for: - children aged 7–14 years: 10–15 mL/day (6.7–10.0 g/day) - children aged 1–6 years: 5–10 mL/day (3.3–6.7 g/day) - infants aged <1 year: up to 5 mL/day (up to 3.3 g/day).
HE in adults	•Starting dose of 30–45 mL (20–30 g) three to four times daily; the maintenance dose may be adjusted to achieve two to three soft stools each day.

*HE, hepatic encephalopathy*.

**Table 2:** Beneficial effects of prebiotics.

**Beneficial effect**	**Description**
Altered GM composition	Prebiotics stimulate growth of health-promoting GM species belonging to the genera *Bifidobacterium* and *Lactobacillus* (14)
Enhanced colonization resistance against harmful gut bacteria	Growth of *Bifidobacterium* and *Lactobacillus* populations enhances colonization resistance against pathogens such as *Escherichia coli, Clostridium* (e.g., *C. perfringens), Salmonella*, and *Campylobacter* (14–20) Colonization resistance occurs through increased competition for nutrients and normal colonization sites and increased production of endogenous antimicrobial substances that create an inhospitable environment for pathogen growth (15) Inhibiting the growth of these potentially gastroenteritis-causing pathogens thereby increases host resistance to infection (15)
Formation of favorable metabolites	Fermentation of carbohydrates (i.e., prebiotics) by gut bacteria produces SCFAs such as acetate and butyrate, which are associated with several beneficial effects (8, 14) SCFAs are an important respiratory substrate for intestinal epithelial cells, strengthen the gut barrier function, and modulate the immune response (21–23); SCFAs may play a key role in the prevention and treatment of metabolic syndrome, bowel disorders, and CRC (21, 23, 24); SCFAs may help to maintain intestinal homeostasis by lowering the gut pH to a level below that at which pathogens are able to compete effectively (15)
Increased mineral absorption	Prebiotics stimulate absorption of minerals such as Ca, magnesium, zinc, and iron (10, 25) This effect is attributed mainly to luminal acidification by SCFAs, which increases the solubility of minerals, thereby facilitating their absorption through the gut wall; however, several other mechanisms may also be involved (8–10)
Enhanced gut immunity	The GM plays an important role in mediating immune responses at mucosal surfaces; prebiotics can therefore modulate GI immunity (26)
Protection against inflammation-mediated pathologies	Alterations in the normal GM have been implicated in various inflammation-mediated pathologies, including allergic asthma, obesity, type 2 diabetes, Parkinson disease, rheumatoid arthritis, osteoarthritis, and OP (27, 28) Prebiotic-modulated GM may therefore protect against such inflammation-mediated pathologies (27, 28)
Increased mucosal integrity	Prebiotics may help to protect the integrity of the intestinal mucosal barrier; increased crypt depth through stimulation of cellular proliferation, increased villous height, and increased mucin release have been observed after prebiotic intake (9, 18)
Maintenance of bone health	Prebiotics increase Ca absorption (10, 25) The GM has a central role in maintaining bone health and influences bone turnover and density (29) The anti-inflammatory actions of prebiotics may be of particular relevance in the context of bone health, because inflammation disrupts the bone remodeling cycle, leading to bone loss (30) Evidence from animal studies suggests that prebiotics attenuate bone loss through a reduction in systemic inflammation, thereby potentially protecting against age- or menopause-related OP (31–33)

*Ca, calcium; CRC, colorectal cancer; GI, gastrointestinal; GM, gut microbiota; OP, osteoporosis; SCFA, short-chain fatty acid*.

Although the ability of lactulose to stimulate the growth of beneficial gut bacteria has been known for over 60 years (3, 4), lactulose is not commonly recognized as a prebiotic. This review provides an overview of the preclinical and clinical evidence showing that low-dose lactulose confers a health benefit as it:

- stimulates the growth of health-promoting gut bacteria (e.g., *Bifidobacterium* and *Lactobacillus* spp.)
- inhibits the growth of pathogenic bacteria (e.g., certain clostridia)
- increases the production of beneficial metabolites [e.g., short-chain fatty acids (SCFAs)]
- improves mineral absorption
- and has good overall tolerability.

Implications of these data for the use of lactulose as a prebiotic are also discussed.

### Mechanism of Action of Lactulose

The treatment effects of lactulose arise from its effects on the gut, namely alteration of colonic microbiota and formation of favorable metabolites (e.g., SCFAs) (Figure 2) (4). The human small intestine lacks the enzyme necessary to split the disaccharide lactulose into its component monosaccharides; lactulose, therefore, reaches the large intestine largely intact (1). Once in the colon, lactulose is selectively metabolized by resident colonic microbiota (11), producing SCFAs, intestinal gas (hydrogen, carbon dioxide, and methane) and resulting in increased bacterial mass (1, 11, 34, 35). The ratio of SCFAs produced will be determined by the composition of the host microbiota, as well as the type and quantity of fermentable substrate, pH of the gut, and factors that influence SCFA absorption from the intestine (36).

Acetate, propionate, and butyrate represent the major SCFAs found in the human colon (35). Acetate is the main SCFA produced by fermentation of lactulose (37, 38). Although neither bifidobacteria nor lactobacilli directly produce butyrate upon lactulose fermentation, cross-feeding occurs among the gut microbiota to generate butyrate; members of the genera *Bifidobacterium* and *Lactobacillus* produce acetate and lactate, which are then converted to butyrate by other members of the gut microbiota (39–41).

SCFAs are rapidly absorbed by the colonic epithelium, where they act as substrates for respiration (35), and represent the main carbon flow from the diet through the microbiome to the host (42, 43). Butyrate is the main/preferred source of energy for colonocytes (44, 45). Beyond being fuel for colonocytes, SCFAs have diverse roles in host health, including regulating cells of the immune system, energy storage/metabolism, and gut barrier function (46).

SCFA receptors include G protein-coupled receptors (GPCRs) such as GPR43, GPR41, GPR109A, and OLFR78 (46). SCFAs and their receptors have several benefits in inflammation; interactions between SCFAs and GPCRs expressed in the gut epithelium and immune cells induce mechanisms that play a key role in maintaining homeostasis in the gut and other organs (47). Acetate has been shown to play an important role in the regulation of inflammation in inflammatory and metabolic diseases and in preventing enteric infection (48, 49). Inflammation is also a major risk factor for cancer development in the digestive tract, and it has been shown that SCFAs, including acetate working through GPR43, act to suppress the development of colorectal cancer (CRC) (46, 50).

SCFAs also have a key role in maintaining a healthy and properly functioning mucosa (37), which is important for nutrient absorption. A properly functioning gut barrier is also vital for preventing the translocation of proinflammatory microbial cell wall components (37). Butyrate has a key role in regulating gut permeability, primarily via orchestration of tight junction proteins (37). Butyrate is also known to induce mucin production, which creates a physical barrier between the colonic microbiota and colonic epithelial cells (51).

Production of acids (e.g., SCFAs) via lactulose fermentation results in a lowering of colonic pH (35). Lowering the gut pH to a level below that at which pathogens are effectively able to compete may help to maintain intestinal homeostasis and to prevent infection (15). An acidic environment also increases the solubility of minerals such as calcium (Ca) and magnesium (Mg) salts, which may represent another means by which lactulose enhances the absorption of these minerals from the gut (8–10).

Use of lactulose as a detoxifying agent for HE mainly stems from the ability of lactulose to alter the gut microbiota to decrease ammonia production and absorption (35). Lactulose also acidifies the colonic content so that ammonia present in the blood diffuses into the colon; here, it is converted into ammonium ions and/or incorporated into microbial biomass and is then excreted via the feces (1, 52). Repression of pathogen colonization with lactulose is also thought to occur from the proliferation of health-promoting gut bacteria and the subsequent competitive effects resulting from their occupation of colonization sites (15).

Growth of resident colonic microbial populations leads to a rise in bacterial biomass (35), and *in-vivo* observations have shown higher fecal bacterial biomass to be associated with shorter intestinal transit times (53). Greater stool volume promotes intestinal peristalsis, accelerating the passage of stool through the colon (1). Increased stool volume may also be achieved via a higher fecal moisture content; metabolism of lactulose increases the osmolality of the intestinal contents (6), exerting an intraluminal osmotic effect and increasing water retention in the lumen (1).

### Dose-Dependent Effects of Lactulose

**Figure 3:**
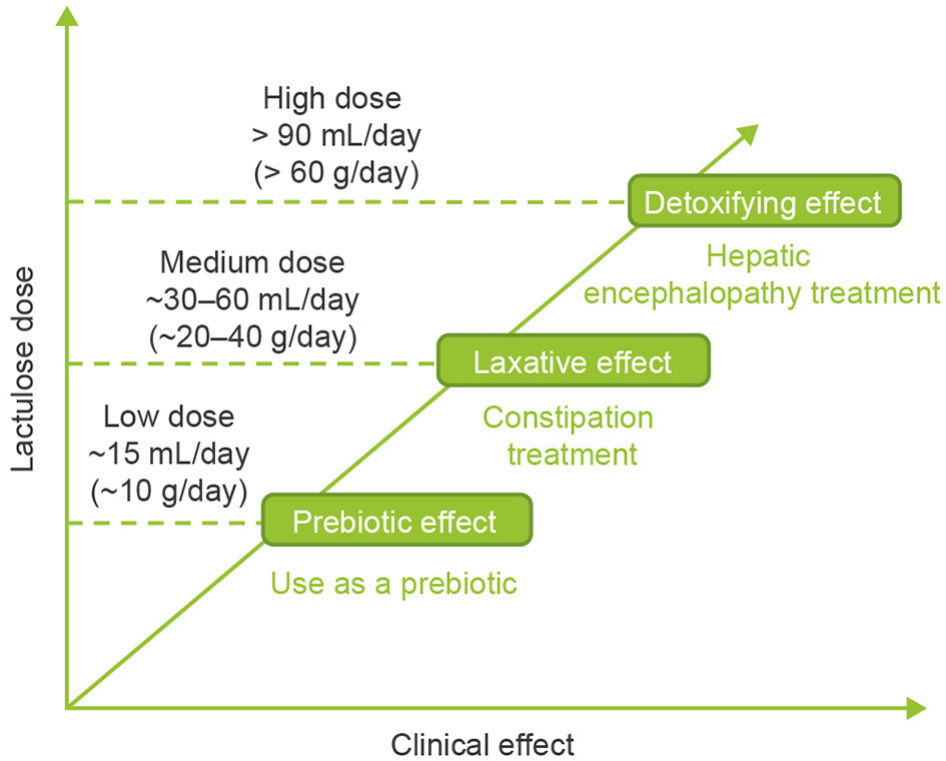
Dose-dependent effects of lactulose (1).

The effects of lactulose are dependent on dose. Depending on the dose used, lactulose can act as a prebiotic, a laxative, or a detoxifying agent (Figure 3) (1). Low doses of ~15 mL/day (~10 g/day; adult dose) produce a prebiotic effect and enhance Ca and Mg absorption, whereas medium doses of ~30–60 mL/day (~20–40 g/day) elicit a laxative effect (used for constipation) and high doses of >90 mL/day (>60 g/day) have a detoxifying effect (used for HE) (1, 4). It is not clear whether these effects are mutually exclusive; concomitant prebiotic and laxative effects of high-dose lactulose have been demonstrated in patients with chronic idiopathic constipation (54).

## Literature Search Methods

To identify relevant studies of the prebiotic effects of low-dose lactulose, a literature search of the PubMed database was conducted with relevant criteria and a cut-off date of August 31, 2020. Search engines used were EMBASE, Google, MEDLINE, Allied and Complementary Medicine, Analytical Abstracts, BIOSIS Previews, and China/Asia On Demand. A manual search of relevant journals was also performed. A broad search string was used: “lactulose” OR “Duphalac” OR “Bifiteral” OR “Betulac” OR “Lactecon” OR “Avilac” OR “Laktipex” AND “prebiotic” OR “bifidogenic.” All publications identified by the search were subsequently reviewed for relevance to the research topic.

## Evidence of the Prebiotic Effects of Low-Dose Lactulose

### Preclinical Evidence

*In vitro*, lactulose was a better carbon source than either lactitol or lactose for the major species of intestinal bacteria (55). Lactulose also dose-dependently increased counts of beneficial gut bacteria (including *Bifidobacterium* and *Lactobacillus*) and levels of SCFAs *in vitro* (56). After 120 h, the mean (± standard deviation [SD]) amount of total SCFAs produced with 2, 3, 4, and 5 g/day lactulose was 451 (± 3) mmol, 399 (± 21) mmol, 427 (± 76) mmol, and 471 (± 12) mmol, respectively, compared with 332 (± 34) mmol with control (56). A study in C57BL/6J mice showed that high- and low-dose lactulose increased SCFA production in the intestine, but concentrations differed according to intestinal site and no statistical differences were seen for the main SCFA in feces (57). Interestingly, acetate concentrations were higher in the animals fed with low-dose lactulose at all intestinal sites and in feces, but only statistically significant in the middle colon. Another study in the same mouse model did not show a difference in fecal SCFA when comparing animals fed with high-dose lactulose with control animals, although it did demonstrate a reduction in branched-chain fatty acids in the lactulose-fed group (58). This illustrates the need to carefully consider data from fecal measurements of SCFA, given that concentrations change along the intestinal tract and that SCFA production can be limited by factors other than availability of fermentable substrate. In both studies, lactulose modulated the gut microbiota, increasing the abundance of bifidobacteria and akkermansiae in particular.

### Clinical Evidence

Nine clinical trials assessing the prebiotic effects of low-dose lactulose were identified, including a total of 537 participants (16, 54, 59–65). All but two of the studies were conducted in healthy volunteers; 69 of the 304 participants in one study could be considered as having mild constipation (60), and another study was conducted exclusively in individuals with chronic idiopathic constipation (*n* = 65) (54). The trend across the studies was for administration of low-dose lactulose to increase populations of beneficial gut bacteria (e.g., *Bifidobacterium* and *Lactobacillus* spp.) and metabolites (e.g., SCFAs), to reduce harmful gut bacteria (e.g., certain clostridia) and to lower fecal pH (Table 3).

**Table 3:** Summary of key efficacy findings from clinical studies of the prebiotic and mineral absorption effects of low-dose lactulose.

**Study population, age**	***N***	**Aims**	**Design**	**Treatment regimen**	**Key efficacy findings**	**References**
**Prebiotic effects**						
Healthy M and F 8–22 y	8	Assess the effects of lactulose on the composition and metabolic activity of fecal microbiota	Open-label, single-arm study	Lactulose 3 g/D for 2 W	↑ (~7%) in populations of bifidobacteria (*p* < 0.001) ↓ (slight) in populations of Bacteroidaceae and lecithinase-positive clostridia (both *p* < 0.05) ↓ fecal indole, phenol (both *p* < 0.05), and skatol (in 4/8 volunteers) ↓β-glucuronidase, nitroreductase, and azoreductase activities (all *p* < 0.05) ↓ fecal pH 7.0 → 6.4 ↑ fecal moisture content by 4.3–5.3%	(63)
Healthy^a^ M and F 13–66 y	304	Assess the effects of lactulose on intestinal function and fecal character	Open-label study in three groups	*n* = 8 healthy volunteers: lactulose 4 g/D for 3 W *n* = 296^a^: lactulose 3 or 5 g/D for 10 D	Lactulose 4 g/D: ↑ populations of bifidobacteria (*p* < 0.05) ↓ populations of Bacteroidaceae, eubacteria, and clostridia (mean ratio to total bacteria when compared with after lactulose intake; all *p* < 0.05) ↓ fecal pH (compared with after lactulose intake; *p* < 0.05) ↓ fecal indole (compared with before lactulose intake; *p* < 0.05) Lactulose 3 or 5 g/D: ↑ defecation frequency and feces became more watery, yellowish, and softer (compared with before and after lactulose intake for both 3 and 5 g/D groups; *p* < 0.05 or *p* < 0.01, respectively) Results were consistent between patients with low and normal defecation frequencies	(60)
Healthy^b^ 18–50 y	20	Assess the effects of lactulose on colonic microbiota	Randomized, double-blind, PBO-controlled study	R 1:1 to lactulose 10 g/D (*n* = 10) or PBO (glucose/lactose; *n* = 10) for 26–33 D	With lactulose: ↑ populations of *Bifidobacterium* spp. vs. pre-tx levels (*p* < 0.01) ↑ populations of *Bifidobacterium* spp. vs. PBO (*p* < 0.01) ↓ populations of *Clostridium* spp. vs. pre-tx levels (*p* < 0.01)	(64)
Healthy M and F 19–42 y	16	Assess the effects of prolonged lactulose on fecal bifidobacteria and metabolic indices potentially involved in colonic carcinogenesis	Randomized, double-blind, PBO-controlled, parallel-group study	R 1:1 to lactulose 10 g/D (*n* = 8) or PBO (sucrose; *n* = 8) for 6 W	With lactulose: ↑ fecal *Bifidobacterium* counts from D0 to D21 and D42 (*p* = 0.048) and vs. PBO (*p* = 0.03) Neither group showed significant changes in total anaerobes, lactobacilli, pH, or other study variables	(59)
Healthy^b^ 24–31 y	36	Assess the comparative efficacy of lactulose and lactitol on colonic microbiota and fecal biochemistry	Randomized, double-blind, PBO-controlled study	R 1:1:1 to lactulose 20 g/D (*n* = 12), lactitol 20 g/D (*n* = 12), or PBO (sucrose/lactose; *n* = 12) for 4 W	Lactulose vs. PBO: ↑ populations of probiotic bacteria (*p* < 0.01) ↓ populations of putrefactive bacteria (*p* < 0.01) Beneficial changes greater with lactulose vs. lactitol Effect onset more rapid with lactulose vs. lactitol (1 vs. 2 W) Both lactulose and lactitol led to significant changes in fecal biochemistry compared with PBO	(16)
Healthy postmenopausal F 55–64 y	10	Assess the effects of lactulose on intestinal microbiota and SCFA production	*In-vivo* effect on fecal samples and computer-controlled *in-vitro* model of the proximal large intestine	Lactulose 10 g/D for 7 D. Microbiota obtained from volunteers before and after lactulose consumption were adapted to an *in-vitro* model of the proximal colon and then fed lactulose 10 g/D introduced gradually over a 48-h period	Following *in-vivo* lactulose consumption: no changes in fecal pH, dry weight, or mean molar SCFA ratios in the fecal samples ↑ populations of *Bifidobacterium* (*p* < 0.05) Following adaptation of the *in-vivo* samples (before and after lactulose consumption) to the *in-vitro* culture system:clear effect of *in-vivo* lactulose consumption on *Lactobacillus* and *Enterococcus* (both ↑) clear effect of *in-vivo* lactulose consumption on SCFA ratios ( ↓ butyrate; *p* < 0.001)	(65)
Healthy F 18–21 y	26	Assess the prebiotic effects of lactulose on defecation frequency	Open-label, single-arm, before-after study	Lactulose 1, 2, and 3 g/D for 2 W each. Crossed over after a 2-W washout period	Lactulose 1, 2, and 3 g/D: ↑ defecation frequency ↑ defecation D ↑ fecal bifidobacteria counts	(61)
Healthy F mean (± SD): 20.2 (± 2.4) y	52	Assess the prebiotic effects of lactulose on defecation frequency	Randomized, double-blind, PBO-controlled, crossover study	R 1:1 to lactulose 2 g/D or PBO (glucose) for 2 W. Crossed over after a 3-W washout period	Lactulose vs. PBO: ↑ populations of *Bifidobacterium* in feces ↑ proportion of *Bifidobacterium* in feces ↑ defecation frequency ↑ number of defecation D improved fecal character (consistency and volume)	(62)
Chronically constipated M and F mean (± SD): 57 (± 18) y	65	Assess the comparative efficacy of lactulose and PEG-4000 on colonic microbiota	Prospective, multicenter, randomized, single-blind, active-controlled, parallel-group study	R 1:1 to lactulose or PEG-4,000 for 4 W. W1 dose fixed (20 g/D); W2 dose could vary (10–30 g/D); W3–4 dose fixed (10–30 g/D)	With lactulose (D −1 to D28): ↑ populations of fecal bifidobacteria and anaerobes (*p* < 0.02) no significant differences in SCFAs With PEG-4000 (D−1 to D28): no significant differences in populations of fecal bifidobacteria/anaerobes ↓ total SCFAs (*p* = 0.02), acetate (*p* = 0.02), and butyrate (*p* = 0.04)	(54)
**Mineral absorption effects**						
Healthy M 23–42 y	24	Assess the effect of lactulose on Ca and Mg absorption	Randomized, double-blind, three-period, three-group crossover study	R 1:1:1 to PBO, lactulose 2 or 4 g/D, plus CaCO_3_ 300 mg (20 mg ^44^Ca) and MgO 150 mg (28 mg ^25^Mg). Crossed over after a 2-W washout period between each tx	Lactulose 2 or 4 g/D enhanced Ca and Mg absorption vs. PBO Urinary stable isotopes ratios (^44^Ca/^40^Ca and ^25^Mg/^24^Mg) ↑ with lactulose dose and were significantly different for lactulose vs. PBO for Ca (lactulose 4 g/D) and Mg (lactulose 2 and 4 g/D) (all *p* < 0.01)	(66)
Healthy postmenopausal F 56–64 y	12	Assess the effect of lactulose on Ca absorption	Randomized, double-blind, PBO-controlled crossover study	R 1:1:1 to lactulose 5 or 10 g/D or PBO (aspartame) for 9 D. ^44^Ca and ^48^Ca given on D8. Crossed over after a 19-D washout period between each tx	Lactulose 5 or 10 g/D dose-dependently ↑ intestinal Ca absorption without ↑ urinary excretion	(67)
Postmenopausal, with osteopenia F 52–67 y	41	Assess the effect of lactulose on BMD maintenance	Randomized, double-blind, PBO-controlled parallel-group study	R 1:1 to lactulose 10 g/D plus CaCO_3_ 500 mg/D or PBO plus CaCO_3_ 1,000 mg/D for 12 months	Lactulose plus CaCO_3_ 500 mg/D was as effective as lactulose plus CaCO_3_ 1,000 mg/D	(68)

a *69 patients could be considered mildly constipated (defecation frequency < 1.0/D)*.

b *Sex of participants not stated*.

*↓, decreased; ↑, increased; BMD, bone mineral density; Ca, calcium; D, day(s); F, female; M, male; Mg, magnesium; n, number of participants; N, total number of participants; PBO, placebo; PEG-4000, polyethylene glycol-4000; R, randomized; SCFA, short-chain fatty acid; SD, standard deviation; spp., species (plural); tx, treatment; W, week(s); y, years*.

In an open-label, single-arm study, eight healthy volunteers received a once-daily drink containing 3 g of lactulose for 2 weeks, in addition to their normal diet (63). During the lactulose intake period, the number of bifidobacteria increased significantly compared with values before intake; mean (± SD) log_10_ cells/g feces was 9.7 (± 0.1) before treatment (day 0) compared with 10.4 (± 0.1) log_10_ cells/g feces on day 7 of intake. Conversely, the numbers of lecithinase-positive clostridia, including *Clostridium perfringens*, and Bacteroidaceae decreased slightly but significantly compared with values before intake (63). After 7–14 days of treatment, lactulose also significantly reduced the levels of potentially toxic substances, including fecal indole and phenol, and significantly reduced activities of fecal β-glucuronidase, nitroreductase, and azoreductase. Finally, lactulose contributed to improvements in the intestinal environment; by day 14 of intake, mean fecal pH decreased from 7.0 to 6.4 and mean water content increased by 3.5–5.3% (63).

**Figure 4:**
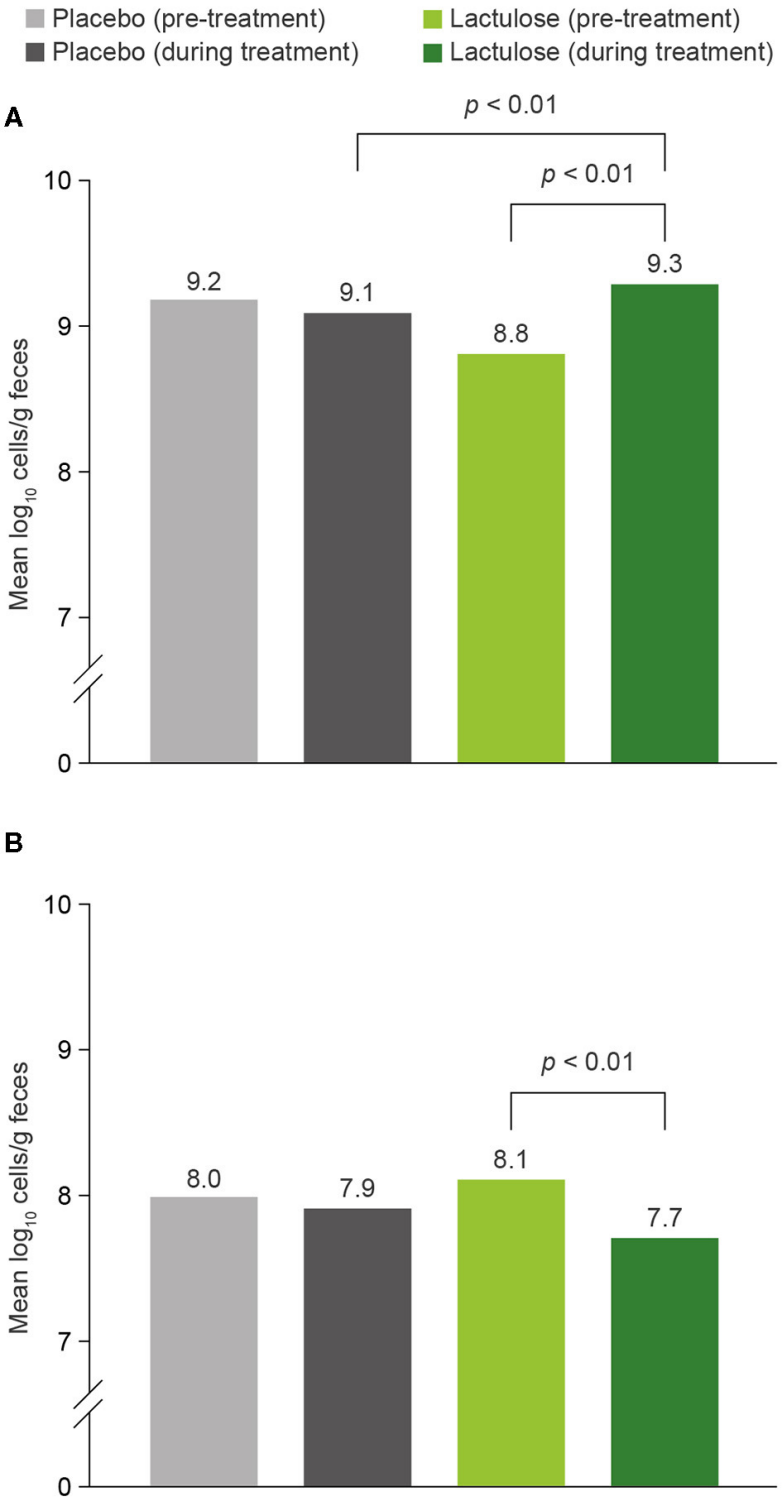
Impact of lactulose 10 g/day and placebo on **(A)** *Bifidobacterium* and **(B)** *Clostridium* counts (assessed by FISH) (64). *n* = 20. FISH, fluorescence *in-situ* hybridization.

In a placebo (PBO)-controlled randomized clinical trial (RCT), 20 healthy volunteers received either lactulose 10 g/day or glucose/lactose (PBO) for between 26 and 33 days (64). Lactulose significantly increased populations of *Bifidobacterium* spp. compared with pre-treatment levels; mean (± SD) log_10_ cells/g feces was 8.8 (± 0.5) before treatment compared with 9.3 (± 0.3) log_10_ cells/g feces after treatment (Figure 4A). This increase was also significant compared with the changes in *Bifidobacterium* spp. population levels that occurred with PBO over the same period. The effect was most pronounced in individuals with the lowest pre-treatment *Bifidobacterium* spp. population counts. There was a significant reduction in levels of *Clostridium* spp. during lactulose intake, from 8.1 (± 0.5) log_10_ cells/g feces before treatment to 7.7 (± 0.4) log_10_ cells/g feces after treatment (Figure 4B). No significant differences in population levels of *Clostridium* spp. were observed in the PBO group over the treatment period or between the lactulose and PBO groups (64).

A parallel-group, PBO-controlled RCT was carried out to assess the effects of prolonged low-dose lactulose on fecal bifidobacteria (59). Sixteen healthy volunteers were randomized to lactulose 10 g/day or sucrose (PBO) for 6 weeks. Fecal bifidobacterial counts were significantly higher after prolonged low-dose lactulose ingestion than after PBO ingestion. Lactulose led to significantly increased fecal *Bifidobacterium* counts from days 0 to 21 and day 42 [mean ± standard error of the mean, 8.25 ± 0.53, 8.96 ± 0.40, and 9.54 ± 0.28 log colony-forming units (CFU)/g wet weight, respectively] (59). Throughout the study, total anaerobes, *Lactobacillus* spp., pH, and other variables did not change significantly in either group (59).

In another RCT, 36 healthy volunteers were randomized to either lactulose 20 g/day, lactitol 20 g/day, or sucrose/lactose (PBO) for 4 weeks (16). Lactulose and lactitol significantly increased populations of *Bifidobacterium, Lactobacillus*, and *Streptococcus* spp. by 3.0, 1.9, and 1.2 log CFU, and 1.4, 0.7, and 0.6 log CFU, respectively. Lactulose and lactitol significantly decreased populations of *Bacteroides* spp., *Clostridium* spp., coliforms, and *Eubacterium* spp. by 4.1, 2.3, 1.8, and 3.0 log CFU, and 1.5, 1.2, 1.0, and 1.9 log CFU, respectively (16). Beneficial changes were greater with lactulose than with lactitol, and the onset of effect was more rapid with lactulose (1 vs. 2 weeks with lactitol) (16). Lactulose and lactitol both led to significant changes in fecal biochemistry (pH, fecal moisture, and SCFAs) compared with PBO (16).

An open-label, single-arm, “before-after” study in 26 healthy Japanese women consisted of a pre-observation period followed by three 2-week ingestion periods with a 2-week washout period between each (61). Across the three ingestion periods, volunteers received escalating doses of lactulose (1, 2, and 3 g/day). Compared with the pre-observation/washout periods, fecal bifidobacterial counts, defecation frequency, and number of defecation days significantly and dose-dependently increased following intake of lactulose 1, 2, and 3 g/day (61). The mean (± SD) number of bifidobacteria significantly increased from 9.93 (± 0.57) log CFU/g feces to 10.10 (± 0.40) log CFU/g feces with lactulose 1 g/day, from 9.95 (± 0.63) log CFU/g feces to 10.23 (± 0.53) log CFU/g feces with lactulose 2 g/day, and from 10.09 (± 0.51) log CFU/g feces to 10.38 (± 0.28) log CFU/g feces with lactulose 3 g/day. These results suggest that doses of lactulose as low as 1 g/day can exert a prebiotic effect (61).

The same study team conducted a crossover RCT in 52 healthy Japanese women (62). Volunteers were randomized to lactulose 2 g/day or glucose (PBO) for 2 weeks. After a 3-week washout period, participants were crossed over to the other treatment group. The mean (± standard error) number of bifidobacteria in feces was significantly higher with lactulose compared with PBO [9.53 (± 0.06) vs. 9.16 (± 0.06) log CFU/g feces, respectively] (62). The proportion of *Bifidobacterium* spp. in feces was also significantly higher after lactulose than after PBO treatment [25.3% (± 1.4%) vs. 18.2% (± 1.4%)]. Moreover, lactulose administration also increased defecation frequency and the number of defecation days, and improved fecal consistency compared with PBO (62).

The only study conducted in postmenopausal women compared the effect of lactulose on fecal parameters *in vivo* with the effect in an *in-vitro* model of the proximal large intestine (65). Fecal samples from 10 healthy postmenopausal volunteers were collected before and after 7 days receiving lactulose 10 g/day. In the *in-vitro* model, lactulose 10 g/day was fed to microbiota over a 48-h period (65). Lactulose promoted *Bifidobacterium* growth *in vivo* and *Lactobacillus* and *Enterococcus* spp. growth *in vitro* (65). No changes in fecal pH, dry weight, or mean molar SCFA ratios were observed in the *in-vivo* fecal samples. However, there was a clear effect on SCFA ratios in the *in-vitro* model, with lactulose causing a pronounced reduction of butyrate by the postmenopausal microbiota (65). The authors concluded that the *in-vitro* model provided a better reflection of the effects of lactulose fermentation in the proximal colon in terms of microbial composition changes and metabolite production, and that, *in vivo*, feces do not closely reflect proximal colon fermentation but a summation of microbiota-related activities from proximal to distal colon (65).

An open-label study consisted of 304 Japanese volunteers split across three lactulose dose groups (60). In the first group, eight healthy volunteers received lactulose 4 g/day for 3 weeks. The remaining 296 participants were divided into two groups, distributed evenly with respect to age and sex, and received either lactulose 3 or 5 g/day for 10 days (60). Of the 296 participants who received lactulose 3 or 5 g/day, 69 had low stool frequency and could therefore be considered as having mild constipation (60). At a dose of 4 g/day, lactulose significantly increased bifidobacterial populations; the ratio of bifidobacteria to total bacteria increased from 22.4% before lactulose intake to 50.5% during intake. Corresponding Bacteroidaceae, eubacteria, and clostridia populations decreased significantly; the proportion of Bacteroidaceae, for example, decreased from 48.4% before lactulose treatment to 28.8% after treatment (60). At 4 g/day, lactulose significantly increased defecation frequency (0.83/day before intake vs. 0.95/day during intake), reduced fecal pH (6.33 during intake vs. 6.52 after intake; no significant difference during intake vs. before intake) and reduced fecal indole (70.3 μmol/g feces before intake vs. 38.7 μmol/g feces during intake). At 3 or 5 g/day, lactulose resulted in a significant increase in defecation frequency and the feces became more watery, yellowish, and softer. Results were consistent between individuals with low defecation frequency and those with normal defecation frequency (60).

Finally, a single-blind RCT compared the effect of lactulose with that of another osmotic laxative, polyethylene glycol 4000 (PEG-4000) on colonic microbiota. This was the only active-comparator RCT identified and the only study conducted in individuals with chronic constipation (*n* = 65) (54). The diagnosis of chronic idiopathic constipation was based on the Rome I diagnostic criteria of constipation: the presence for at least 6 months of fewer than three stools per week and/or difficulty in defecation and/or straining on passage of stool (54). Lactulose or PEG-4000 was given at a dosage of 20 g/day for the first week. During week 2, dose adjustments were permitted depending on the efficacy and tolerance of lactulose (allowing a dose of 10–30 g/day) (54). Following dose adjustment, the investigator fixed the dose for the last 2 weeks. From days −1 to 28, median fecal bifidobacteria and anaerobe counts increased significantly with lactulose (from 8.4 to 9.1 log CFU/g wet weight and from 10.6 to 10.9 log CFU/g wet weight, respectively), whereas no significant changes were observed with PEG-4000 (54). Over the same time period, metabolic activity of fecal microbiota was strongly inhibited with PEG-4000; there was a significant decrease in levels of total SCFAs, butyrate, and acetate. No significant differences in levels of SCFAs were noted with lactulose (54), and no differences were seen in either treatment group in fecal pH or in fecal counts of *Lactobacillus*, clostridial spores, *Bacteroides*, or enterobacteria (54).

Taken together, the results of clinical studies published to date, consistent with preclinical data, show that low-dose lactulose increases counts of *Bifidobacterium* and *Lactobacillus* spp. and beneficial SCFAs, reduces the growth of harmful gut bacteria (e.g., certain clostridia), and lowers fecal pH.

## Evidence of the Mineral Absorption Effects of Low-Dose Lactulose

### Preclinical Evidence

Preclinical studies have shown that lactulose stimulates Ca and Mg absorption from the gut in rats, an effect that appears to occur in both the small intestine and the cecum (25, 69–71).

### Clinical Evidence

In separate clinical studies, low-dose lactulose has been shown to enhance mineral absorption in healthy men (66) and in postmenopausal women (Table 3) (67).

A randomized, double-blind, three-group crossover study was conducted in 24 healthy men (23–42 years old) to evaluate the effect of lactulose on Ca and Mg absorption (66). Volunteers received test food containing lactulose 0 g (PBO), 2 or 4 g together with CaCO_3_ 300 mg (containing 20 mg of ^44^Ca) and MgO 150 mg (containing 28 mg of ^25^Mg). Participants crossed over to each of the other two lactulose doses, with a 2-week washout period between each treatment. Results showed that the higher the dose of lactulose, the higher the urinary stable isotopes ratios (^44^Ca/^40^Ca and ^25^Mg/^24^Mg). This difference was significant for Ca between PBO and lactulose 4 g and for the Mg ratio between PBO and both doses of lactulose. This study demonstrates that low-dose lactulose enhances the absorption of Ca and Mg in healthy men and that it does so in a dose-dependent manner (66).

**Figure 5:**
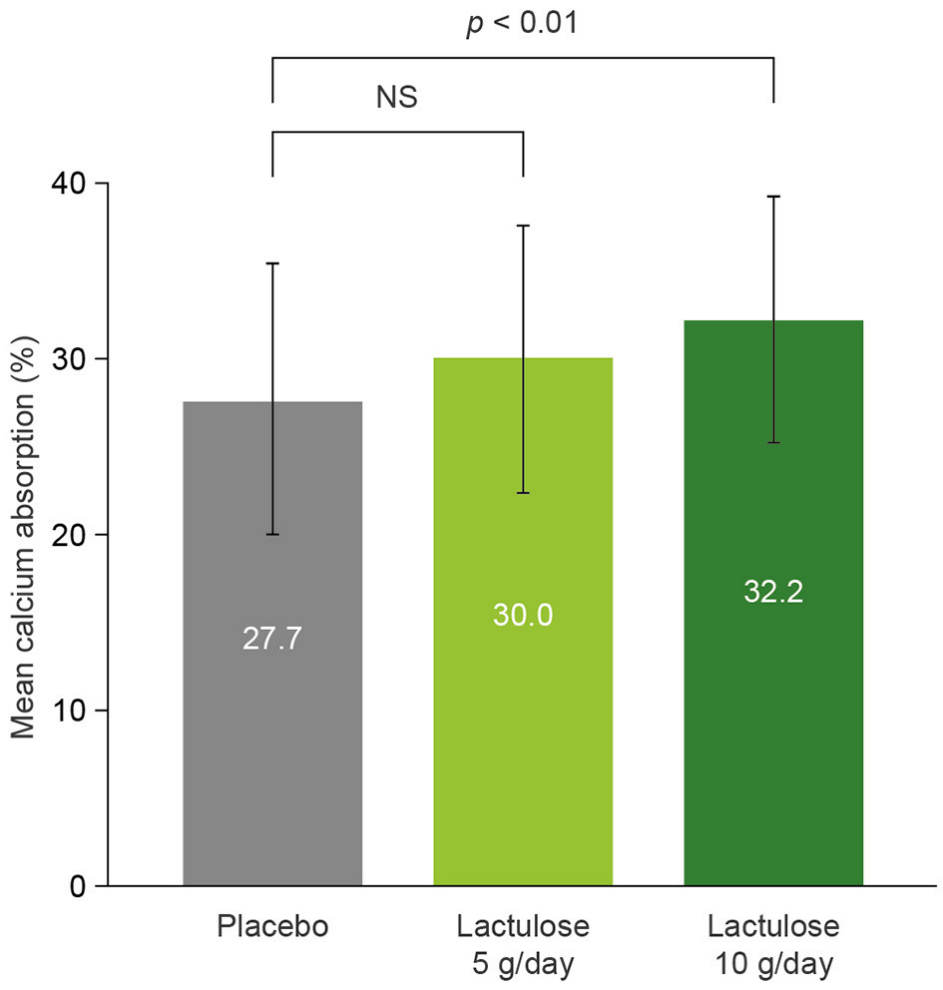
The effect of lactulose on calcium absorption in healthy postmenopausal women (67). *n* = 12. Error bars show ± standard deviation. NS, not significant.

A similar dose-dependent increase in Ca absorption with lactulose was observed in a randomized, double-blind, PBO-controlled crossover study in 12 healthy postmenopausal women (aged 56–64 years) (67). Participants drank 100 mL of water containing lactulose 5 or 10 g or PBO for 9 days. Oral ^44^Ca and intravenous ^48^Ca were administered on day 8 of treatment, and urine isotope measurements were used to calculate Ca absorption. A 19-day washout period separated each treatment. Mean (± SD) Ca absorption with PBO, lactulose 5 g/day, and lactulose 10 g/day was 27.7% (± 7.7%), 30.0% (± 7.6%), and 32.2% (± 7.0%), respectively. The difference in Ca absorption between lactulose 10 g/day and PBO was significant (Figure 5) (67).

The chronic effect of lactulose use on maintenance of bone mineral density (BMD) has also been assessed in postmenopausal women with osteopenia (68). In a randomized, double-blind, PBO-controlled parallel-group study, 41 women received either lactulose 10 g, vitamin D3 400 IU, and CaCO_3_ 500 mg, or PBO, vitamin D3 400 IU plus CaCO_3_ 1,000 mg once daily for 12 months. Baseline daily Ca intake was similar in both treatment arms. Differences in least-square means of BMD (measured in the lumbar spine) between lactulose and PBO at final visit were not statistically significant. The results suggest that lactulose may help to maintain BMD in postmenopausal women by increasing Ca absorption (68).

## Evidence of the Safety Profile of Low-Dose Lactulose

Lactulose is absorbed in insignificant amounts in the gut, which then undergo rapid excretion by the kidneys; the direct effects of lactulose, therefore, remain localized to the gut (72). Of the 11 clinical studies of the prebiotic/mineral-absorption effects of low-dose lactulose included in this review, eight reported safety outcomes [from a total of 519 participants (385 healthy volunteers; 69 with mild constipation, with defecation frequency <1.0/day; 65 with chronic constipation)] (54, 59–62, 64, 66, 67). These studies demonstrated that low-dose lactulose (1–10 g/day) is well-tolerated in healthy adults (including postmenopausal women) and adults with constipation, with few mild to severe abdominal/GI symptoms or other adverse effects (Table 4). GI symptoms seen with lactulose are dose-dependent; the higher the dose, the greater the incidence of symptoms such as abdominal pain, bloating, and diarrhea (73). At the dose relevant for its use as a prebiotic, as per the eight studies that reported safety outcomes, lactulose is generally associated with mild digestive symptoms, such as small increases in flatulence and abdominal distension/bloating (54, 59–62, 64, 66, 67). Furthermore, when GI symptoms do occur, they usually remit spontaneously within a few days of starting treatment or upon dose reduction (72).

**Table 4:** Summary of key safety/tolerability findings from clinical studies of lactulose.

**Design and study population**	**Key safety findings**	**References**
**Prebiotic effects of lactulose**		
Open-label study in 304 healthy adult volunteers (one group non-constipated; one group mildly constipated)	Lactulose was generally well-tolerated at all doses Most participants reported that treatment had no significant tolerability effect (59–80% of all abdominal symptom comments were “nothing significant”) However, small increases in abdominal gaseous symptoms (flatulence, abdominal distension, passing flatus) were observed in both treatment groups	(60)
Randomized, double-blind, PBO-controlled study in 20 healthy adult volunteers	Lactulose 10 g/day was generally well-tolerated One participant reported a moderate to severe change in flatulence, bloating, and accompanying abdominal pain	(64)
Randomized, double-blind, PBO-controlled, parallel-group study in 16 healthy adult volunteers	Prolonged low-dose lactulose (10 g/day) was well-tolerated and was associated with mild digestive symptoms Excess flatus was more common in the lactulose group vs. PBO (*p* = 0.03) but was very mild. Bloating, borborygmi, and abdominal pain did not differ between the groups	(59)
Open-label, single-arm, before-after study in 26 healthy women	Low-dose lactulose (1, 2, and 3 g/day) was well-tolerated No side effects or SAEs were reported Secondary abdominal symptoms were predominantly GI in nature; however, their incidence did not differ significantly between pre-observation/washout periods and respective lactulose intake periods	(61)
Randomized, double-blind, PBO-controlled, crossover study in 52 healthy women	No side effects or SAEs were reported The main tolerability symptoms were GI in nature, but these were similar for low-dose lactulose (2 g/day) and PBO	(62)
Prospective, multicenter, randomized, single-blind, active-controlled, parallel-group study in 65 adults with chronic constipation	The proportion of patients reporting at least 1 day of moderate-to-severe borborygmi and bloating decreased in both the lactulose and PEG-4000 treatment groups Eight patients in the lactulose group and five in the PEG-4000 group reported a total of 17 AEs (events assessed included borborygmi, bloating, abdominal pain, and excess flatus); however, there were no SAEs	(54)
**Mineral absorption effects of lactulose**		
Randomized, double-blind, three-period, three-group crossover study in 24 healthy men	Low-dose lactulose (2 or 4 g/day) was well-tolerated, with no side effects reported	(66)
Randomized, double-blind, PBO-controlled crossover study in 12 healthy postmenopausal women	Low-dose lactulose (5 and 10 g/day) was well-tolerated There were no significant differences in GI complaints between low-dose lactulose and aspartame PBO treatment in a postmenopausal population	(67)

*AE, adverse event; GI, gastrointestinal; PBO, placebo; PEG-4000, polyethylene glycol-4000; SAE, serious adverse event*.

## Discussion

The studies included in this review clearly demonstrate that the prebiotic health benefits of lactulose extend beyond a simple osmotic laxative effect observed at higher doses; evidence shows that low-dose lactulose stimulates the proliferation of *Bifidobacterium* and *Lactobacillus* spp. and the production of SCFAs, reduces levels of harmful gut bacteria (e.g., certain clostridia), and improves mineral absorption. Low-dose lactulose is also well-tolerated, with few mild to severe abdominal/GI symptoms or other adverse effects.

### Extrapolation of the Results to Special Populations

It is important to note that the studies included in this review were conducted mostly in healthy adult volunteers; outcomes in special populations and non-healthy individuals may therefore differ from those reported here. Two studies were conducted exclusively in postmenopausal women (65, 67), and two other studies included patients with constipation (54, 60); however, none included special populations such as women who were pregnant or lactating, children, or the elderly. Nevertheless, when used at higher doses than investigated here (i.e., for chronic constipation or HE), lactulose has demonstrated a favorable safety profile in these populations (74). Similarly, although patients with diabetes were not included in these studies, it has been shown that blood glucose levels remain unchanged after lactulose intake in healthy volunteers, suggesting that lactulose as a functional food ingredient may also be consumed by people with impaired glucose tolerance (75). The effects of lactulose established in healthy individuals cannot, however, be extrapolated reliably to patients with certain diseases, such as irritable bowel syndrome, liver disease (e.g., cirrhosis), and chronic kidney disease (76). Although low-to-medium doses of lactulose (10–30 g/day) were shown to have prebiotic effects in patients with chronic idiopathic constipation (54), there is a notable lack of data on the prebiotic effects of lactulose in GI disorders other than constipation. There is therefore a need for separate studies of the effect of lactulose on the composition of the gut microbiota in patients with different pathologies (76).

### Lactulose Dose Considerations

Given the dose-dependent nature of GI symptoms, the higher the dose of lactulose, the more likely patients are to experience diarrhea (72). Concerning the addition of lactulose to infant formula milk, the incorporation of 0.5% lactulose is considered adequate to stimulate bifidobacterial growth to the extent observed in breast-fed babies, while preventing any laxative action due to lactulose (77). The transitory laxative threshold for lactulose has been estimated to be 0.26 g/kg body weight, which indicates that it would be acceptable to administer lactulose at a dose of up to 13 g/day for a person weighing 50 kg; doses beyond this threshold are more likely to induce diarrhea (78). The European Food Safety Authority recognizes lactulose at a dose of 10 g/day as a food supplement and supports the claim that daily consumption of lactulose at this dose brings about “a reduction in intestinal transit time” (79).

The dose range of 1–10 g/day of lactulose used in the clinical studies included in this review is too broad a range to be practical. We therefore suggest that, for use as a prebiotic in adults, a dose of 5–10 g/day of lactulose is likely to provide a positive benefit–risk ratio while being practical and convenient for the patient.

### Potential Benefits of Low-Dose Lactulose

#### Mineral Absorption and Bone Health

Two studies in this review, including one in healthy postmenopausal women, demonstrated that lower doses of lactulose increase the absorption of minerals from the gut (66, 67). The increased absorption of Ca and Mg with lactulose treatment appears to occur primarily in the small intestine, with some evidence that it may also take place in the cecum (25). Increased absorption of Ca, in particular, may have important implications for maintaining or improving bone density. The bone-health-supporting potential of prebiotics such as lactulose will depend on the host's characteristics, such as their age, postmenopausal status, and capacity to absorb Ca (9). Individuals who have a high demand for Ca (e.g., those who are going through puberty or are postmenopausal) are more likely to benefit from prebiotics than healthy adults (9). During bone development, which typically takes place during adolescence but can continue into early adulthood, BMD increases until peak bone mass is reached (80). Importantly, peak bone mass is a key determinant of osteoporosis later in life (81). Given the critical role of Ca in bone formation and the importance of the increase in BMD that occurs during bone development, lactulose may have a role in ensuring adequate Ca intake during this crucial period.

Because Ca absorption declines with age, older patients could also derive particular benefit from low-dose lactulose treatment (82, 83). In particular, women experience a rapid decline in intestinal Ca absorption with the onset of menopause (82, 84). Declining estrogen levels that occur with menopause lead to increased bone turnover, with resorption exceeding formation (31, 85, 86), resulting in rapid bone loss and risk of menopausal osteoporosis (31). Because bone loss in recently postmenopausal women is largely influenced by a decline in circulating estrogen, women who are beyond menopause by more than 6 years may benefit more from lactulose than women who are recently postmenopausal (9). The potential bone-health-enhancing effects of lactulose and the populations likely to benefit most from increased Ca absorption require further investigation.

Similarly, there is a growing realization that inflammation has a significant influence on bone turnover and increases the risk of osteoporosis and other bone and joint chronic pathologies (67, 87). The potential of SCFAs, especially acetate and butyrate, to regulate inflammatory processes both in the gut and systemically therefore raises the intriguing possibility of managing bone health through prebiotics such as lactulose. Studies in mice have shown that treatment with SCFAs and feeding with a high-fiber diet significantly increase bone mass and prevent postmenopausal and inflammation-induced bone loss (88). SCFAs were identified as potent regulators of osteoclast metabolism and bone homeostasis (88).

#### Potential Role of Lactulose as a Prebiotic

At present, lactulose is available as a medicinal product (at medium and high doses for the treatment of constipation and HE, respectively) and at a low dose as a food supplement. Despite lactulose not being widely recognized as a prebiotic, its prebiotic effects are outlined in the pharmacodynamic section of its prescribing information (7). Asian constipation treatment guidelines also highlight the prebiotic effects of lactulose and state that the ability of lactulose to stimulate the growth of health-promoting bacteria in the human gut could contribute to an improvement in bowel function (74, 89).

As reported in this review, data published to date demonstrate that lactulose at a dose of 5–10 g/day exerts prebiotic effects, contributing to a healthy gut environment and increasing mineral absorption. This appears to support both the preventive and the therapeutic use of low-dose lactulose as a prebiotic to improve gut health and to ensure a guaranteed uptake of Ca. Through its potential bone-health-enhancing effects, low-dose lactulose may have a role in combating age- or menopause-associated osteoporosis. Furthermore, given the potential immune-enhancing effects of prebiotics, low-dose lactulose might also prove a useful dietary additive for individuals genetically predisposed to CRC, as well as for the prevention and treatment of other inflammation-mediated pathologies. Further studies are required to test this hypothesis.

#### Immune Modulatory Potential of SCFAs

GPR43, which is a SCFA GPCR, plays a key role in intestinal inflammatory responses in health and disease (90), and the interactions of SCFAs with GPR43 are pivotal in suppressing/resolving colonic inflammation (91). GPR43 is the pre-eminent receptor for acetate in the intestinal setting, although acetate has been shown to activate other GPCRs, such as GPR41 (90). Acetate, the main SCFA generated from lactulose fermentation, acts via GPR43 to regulate immune function, to enhance the inflammatory response against pathogens in the gut, and to regulate/resolve inflammation elsewhere in the body (48, 49). The systemic action of acetate may have important implications for immune-mediated diseases (e.g., cancer, metabolic syndrome, dementia, and autoimmune diseases) and for bone health.

#### Colorectal Cancer Protection

The modulation of gut microbiota represents a novel strategy for the prevention of CRC and the optimization of its treatment (92). A causal relationship exists between intestinal microbial dysbiosis and CRC pathogenesis, whereby several bacterial species have been identified as contributing to colorectal proliferation (e.g., *Fusobacterium nucleatum, Peptostreptococcus anaerobius*, and enterotoxigenic *Bacteroides fragilis*) whereas others (e.g., *Lachnospiraceae* spp., *Bifidobacterium animalis*, and *Streptococcus thermophilus*) have been found to be depleted in patients with CRC (92). This suggests that these depleted bacteria may exert a protective effect against CRC. The use of prebiotics to stimulate the colonic abundance and activity of these health-promoting bacteria or to achieve a direct anti-inflammatory effect on the gut represents a promising therapeutic strategy (92).

Butyrate has been shown to modulate the expression of genes involved in the defense against oxidative and metabolic stress in primary human colon cells *in vitro* (21, 24). This suggests that butyrate-induced changes in gene expression could protect colon cells from oxidative stress and suppress inflammatory reactions known to increase the risk of CRC (24). An *in-vitro* study in colonic macrophages and dendritic cells demonstrated that signaling via the GPR109A receptor, a receptor for butyrate in the colon, promoted anti-inflammatory properties (93). Further, GPR109A deficiency in mice was shown to promote colon carcinogenesis whereas GPR109A activation suppressed colonic inflammation and carcinogenesis (93). Acetate may also have protective effects against CRC, acting via its receptor GPR43 to regulate the inflammation involved in intestinal carcinogenesis (50, 90).

Thus, through promoting the growth of *Bifidobacterium* and the subsequent positive impact on levels of acetate and butyrate, lactulose could feasibly protect against the development of CRC. It should be noted that the suggested inhibitory effect of SCFAs on cancer is not completely understood and further studies are needed into the effects of lactulose on CRC (65).

### Limitations of the Review

Although the literature search to identify studies of interest was in-depth, a systematic approach was not adopted, and it is therefore possible that not all studies on the prebiotic properties of lactulose have been considered. In addition, studies in the field of prebiotics employ a wide variety of microbiological methodologies, model systems, and bacterial nomenclature in both the preclinical and clinical settings, making direct comparisons between studies challenging.

## Conclusions

The prebiotic properties of lactulose have been known for over 60 years, and a wealth of data from studies published over the past 30 years shows that lactulose at a dose of 5–10 g/day exerts prebiotic effects. Nevertheless, lactulose is not widely used as a prebiotic. These studies have demonstrated the efficacy of low-dose lactulose in stimulating proliferation of *Bifidobacterium* and *Lactobacillus* spp., increasing beneficial SCFAs, especially acetate and (to a lesser degree) butyrate, reducing certain clostridia, and improving mineral absorption, while eliciting few adverse effects. Of note, the immune regulatory effects of acetate (the main SCFA produced by lactulose fermentation) may have important implications for regulating the inflammatory response, important for both controlling infections and reducing the risk of chronic inflammatory conditions, including osteoporosis, gout, and CRC. Furthermore, the ability of lactulose to enhance Ca absorption may have implications for enhancing bone density and bone health, which may be of particular clinical relevance for adolescents, postmenopausal women, and individuals at an advanced age. Further studies are required to establish whether the beneficial effects of lactulose can be seen in patients with various pathologies, and whether therapeutic or preventive use of lactulose may be beneficial in diseases such as osteoporosis and CRC.

## Author Contributions

All authors have contributed substantially to the conception and design of the article, to the analysis and interpretation of the relevant data and literature, and to the drafting and critical revision of the content.

## Conflict of Interest

GJ is an employee of Abbott Product Operations AG, Established Pharmaceuticals Division Headquarters, Allschwil, Switzerland. The remaining authors declare that the research was conducted in the absence of any commercial or financial relationships that could be construed as a potential conflict of interest.

## Publisher's Note

All claims expressed in this article are solely those of the authors and do not necessarily represent those of their affiliated organizations, or those of the publisher, the editors and the reviewers. Any product that may be evaluated in this article, or claim that may be made by its manufacturer, is not guaranteed or endorsed by the publisher.
